# Magnetic Properties of Liquid-Phase Sintered CoFe_2_O_4_ for Application in Magnetoelastic and Magnetoelectric Transducers

**DOI:** 10.3390/s120810086

**Published:** 2012-07-25

**Authors:** Vera Lúcia Othéro de Brito, Stéphanie Alá Cunha, Leonardo Violim Lemos, Cristina Bormio Nunes

**Affiliations:** 1 Instituto de Estudos Avançados, Rodovia dos Tamoios, km 5,5, Putim, São José dos Campos, SP 12228-001, Brazil; E-Mails: scunha@ieav.cta.br (S.A.C.); violim@ieav.cta.br (L.V.L.); 2 Escola de Engenharia de Lorena, Universidade de São Paulo, Polo Urbo-Industrial, Gleba AI-6, Lorena, SP 12600-970, Brazil; E-Mail: cristina@demar.eel.usp.br

**Keywords:** magnetic ceramics, ferrites, magnetostriction, magnetoelastic sensitivity, magnetomechanical sensors, ceramics processing, liquid-phase sintering

## Abstract

Cobalt ferrite is a ferrimagnetic magnetostrictive ceramic that has potential application in magnetoelastic and magnetoelectric transducers. In this work, CoFe_2_O_4_ was obtained using a conventional ceramic method and Bi_2_O_3_ was used as additive in order to obtain liquid-phase sintered samples. Bi_2_O_3_ was added to the ferrite in amounts ranging from 0.25 mol% to 0.45 mol% and samples were sintered at 900 °C and 950 °C. It was observed the presence of Bi-containing particles in the microstructure of the sintered samples and the magnetostriction results indicated microstructural anisotropy. It was verified that it is possible to get dense cobalt ferrites, liquid-phase sintered, with relative densities higher than 90% and with magnetostriction values very close to samples sintered without additives.

## 1. Introduction

Magnetostrictive materials are applicable in magnetoelastic transducers for sensing magnetic fields and mechanical stresses. There are several examples in the literature of applications of magnetostrictive ferrites in magnetic field [1], torque [2], compressive stress [3], and hydrostatic stress [4] sensors.

Magneto-electric (ME) composites combine magnetostrictive and piezoelectric phases to form materials that can convert both magnetic and mechanical energies into electricity. These features make ME composites applicable in magnetic field sensors [5] and also as power sources for wireless sensor nodes [6], because they may generate electricity from ambient vibrations and stray magnetic fields.

The distribution of the magnetic and piezoelectric phases in ME composites may vary. Laminate distribution has the advantage of eliminating charge leakage, however, in the case of ceramic composites, co-firing is challenging [7]. Thus, the study of alternatives to adjust the sintering temperatures of the ceramic phases is very important.

Iron-rare earth alloys, like TERFENOL, are widely used in magnestoelastic transducers due to their giant magnetostriction and high magnetoelastic sensitivity [8]. However, these materials are less corrosion resistant compared to ceramic magnetostrictive materials. Cobalt ferrites are magnetic ceramics that attain lower magnetostriction levels than TERFENOL, but they have a much lower cost and have higher resistivity. Several works have pointed out that they are very promising for mechanical stress sensing [2] and magnetoelectric composites [9].

Sintering of ferrites usually requires temperatures around 1,300–1,450 °C. However, in the fabrication of electronic devices, sometimes sintering temperatures lower than 1,000 °C are required. The use of sintering additives that form a liquid phase during sintering is an alternative for lowering the sintering temperature of ceramics. Additives such as V_2_O_5_ [10] and Bi_2_O_3_ [11] have been successfully used to achieve this objective in ferrites. On the other hand, it is known that the sintering parameters affect the cation distribution in the spinel structure of CoFe_2_O_4_ and that cation distribution has a great influence on magnetostriction [12], making this an issue to be considered when reducing the sintering temperature of this ferrite.

CoFe_2_O_4_ presents the highest magnetostriction value among ferrites, which makes this material suitable for magneto-mechanical sensors. The work from Bhame [13] shows that the grain size/density combination influences the magnetostriction of cobalt ferrites and that the highest values of these parameters tend to be obtained when grains are smaller and samples are denser. The objective of this work was to study the magnetic properties of CoFe_2_O_4_, liquid-phase sintered with Bi_2_O_3_ as additive, evaluating the influence of the sintering method on the microstructure, magnetostriction and magnetoelastic sensitivity of the material.

## 2. Experimental Section

Co_3_O_4_ and Fe_2_O_3_ were used as raw materials for the fabrication of CoFe_2_O_4_. These oxides were manually dry mixed in a mortar and calcined at 850 °C for 4 h. Bi_2_O_3_ was added to the calcined powder in the following molar proportions: 0.15%, 0.25%, 0.35%, and 0.45%. The mixing of the additive was carried out in ethyl alcohol, using an eccentric mill. The ethyl alcohol was removed from the mixture by means of combustion.

Part of the calcined powder was sintered at 950 °C for 6 h and its crystalline structure was evaluated by means of X-ray diffraction (XRD). This experiment was carried out in a Panalytical Empyrean X'Pert PRO MRD diffractometer, with CuKα radiation. The parameters used were: generator voltage: 40 kV; tube current: 30 mA; scan step size: 0.02°. The lattice parameter (a) was calculated, based on the XRD results, and the reference mass density (d_ref_) was calculated using Equation (1) [14]. In Equation (1), M is the molar mass and N is the Avogadro's number:

(1) $${\text{d}}_{\text{ref}}=\frac{8\text{M}}{\text{N}\;{\text{a}}^{3}}$$

The calcined Bi_2_O_3_-added powders were uniaxially pressed into tablets of 8-mm diameter and 2-mm thickness, under 50 MPa pressure. The compacted tablets were subsequently pressed in an isostatic press, under 300 MPa. The tablets were sintered at 900 °C for 3 h and at 950 °C for 3h and the values of the mass densities (d) were measured by means of Archimedes' method. The relative mass density (D) was calculated, having d_ref_ as reference. The compositions with D = 90% were selected to have new samples sintered for 6 h. Only the samples that had D > 90% had their microstructures and magnetic properties analyzed in this work.

For magnetic characterization, samples from the tablets were cut in a cubic shape with sides of approximately 3 mm. The magnetization measurements were made using Vibrating Sample Magnetometer—VSM from Quantum Design. The internal fields (H_int_) were calculated from Equation (2) and the demagnetizing fields (H_d_) were calculated from Equation (3). The magnetization values (M) were taken from the VSM measurements results and the geometrical factor (N_d_) was estimated to be 1/3, based on the samples' shapes and dimensions [15]:

(2) $${\text{H}}_{\mathrm{int}}=\text{H}-{\text{H}}_{\text{d}}$$

(3) $${\text{H}}_{\text{d}}=-{\text{N}}_{\text{d}}\;\text{M}$$

The magnetostriction curves parallel (λ_//_) and perpendicular (λ_⊥_) to the applied field “H” were measured by means of capacitance dilatometry [16]. The magnetoelastic sensitivity curves [∂λ/∂(μ_0_H)] and the H_int_ values that corresponded to points of interest in these curves were calculated. The total magnetostriction at saturation (λ_tot_) was calculated according to (4), by taking the magnetostrictions at saturation [(λ_//_)_s_ and (λ_⊥_)_s_] from the magnetostriction curves:

(4) $$\lambda_{\text{tot}}={\left(\lambda_{⊥}\right)}_{\text{s}}-{\left(\lambda_{//}\right)}_{\text{s}}$$

The microstructures of the samples were analyzed using Scanning Electron Microscopy (SEM) images of polished and thermally etched samples' surfaces and the local compositions were assessed by Energy Dispersive X-Ray (EDX) analysis. Because of the low accuracy of EDX analysis to quantify light elements, it was carried out in a qualitative way, not taking oxygen into consideration.

A CoFe_2_O_4_ sample with 93% relative density was obtained by Lemos [17] and was taken as reference in the present work. The reference sample had been processed in a similar way and sintered at 1,300 °C for 2 h without additives. Its magnetostriction had been evaluated following the same procedure as in this work. However, the magnetization measurement had been carried out on a powder sample.

## 3. Results and Discussion

**Figure 1.**
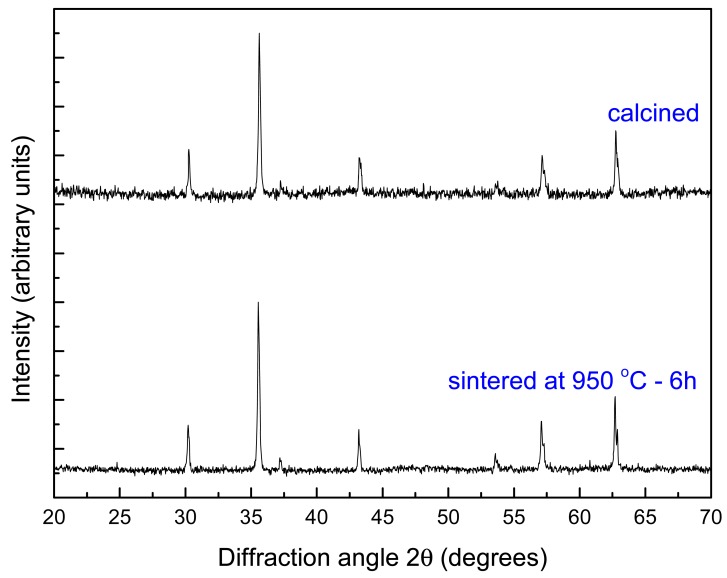
Diffraction patterns of the ferrite calcined and sintered powders.

The results from X-ray analysis of the ferrite powders are shown in Figure 1 and they indicate that both calcined and sintered powders were constituted of the expected spinel phase. The sintered powder had a lattice parameter of 8.37 Å, which is in agreement with experimental data from literature [12]. The calculated “d_ref_” was 5.32 g/cm^3^.

The mass densities obtained by Archimedes' method and the corresponding “D” values of the Bi_2_O_3_-added samples are shown on Table 1. The mass densities varied from 3.76 to 5.11 g/cm^3^, which corresponded to relative densities from 71% to 96%. The samples highlighted in green, which have D > 90%, were the samples selected for magnetic characterization.

**Figure 2.**
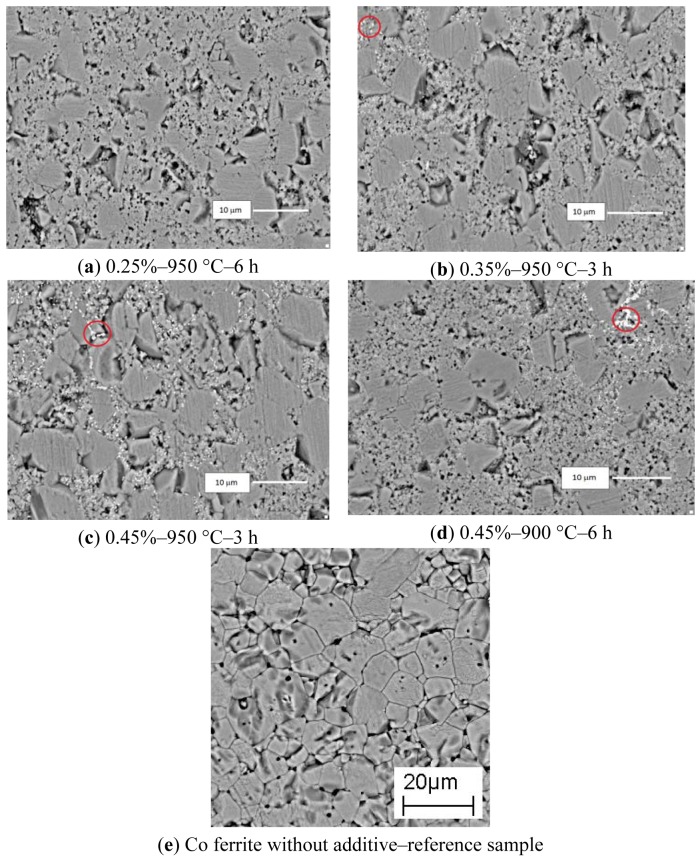
Microstructures of the liquid-phase sintered ferrites (**a–d**) and (**e**) reference sample. The Bi-containing particles are indicated by the red circles.

**Figure 3.**
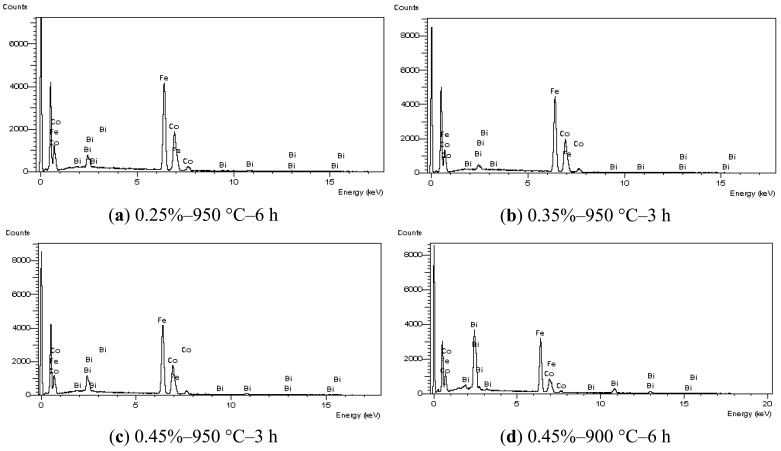
Results of EDX analysis of the Bi-containing particles, not considering oxygen.

The microstructures of the samples that attained D > 90% are shown on Figure 2, together with that of the reference sample. The liquid-phase sintered ferrites presented a non-uniform grain size and the presence of white second-phase particles was observed. The results from EDX analysis, which was carried out in one white particle per sample, are shown on Figure 3. They indicate that these particles contain Fe, Co, and Bi, that probably crystallized from the additive liquid state. The Bi fraction of the particle analyzed in the sample sintered at 900 °C was undoubtedly higher than the fractions found in the samples sintered at 950 °C. In this sample sintered at lower temperature, it is possible that some Bi_2_O_3_, which is a diamagnetic substance [18], remained after sintering.

Jia *et al.* [19] added 3 wt% Bi_2_O_3_ to a Ni-Cu-Zn ferrite and detected, by means of X-ray analysis, traces of the main planes of the BiFeO_3_ phase (bismuth orthoferrite) in the sintered material. Cobalt may substitute iron in this phase, forming BiCo_1-x_Fe_x_O_3_ [20]. The X-ray diffraction pattern of the ferrite with 0.45 mol% sintered at 900 °C during 6 h did not present traces of second phases, but they can be observed in the SEM micrograph (Figure 2(d)).

The observation of the Bi-containing particles in Figure 2 indicates a non-uniform distribution in the microstructure and sometimes it emerges concentrated in some regions, coming out in large sizes. Mürbe and Töpfer [11] studied the microstructure of liquid-phase sintered Ni-Cu-Zn ferrites with Bi_2_O_3_ additions. The authors used high-resolution Transmission Electron Microscopy (TEM) to observe a sample with 0.375 wt% Bi_2_O_3_ addition and sintered at 900 °C for 2 h. They observed the presence of Bi-rich phases forming very thin films between the ferrite grains. It is also possible that Bi^3+^ ions of the additive substitutes Fe^3+^ ions of the ferrite [21], locally altering its stoichiometry to CoFe_2-x_Bi_x_O_4_. Therefore, based on these results, although Bi-containing particles were rarely detected in the micrographs of the sample with 0.25% Bi_2_O_3_, it is expected that higher resolution techniques as Field Emission Gun (FEG) or Transmission Electron Microscopy (TEM) could probably be able to resolve more particles in a nanoscale.

**Figure 4.**
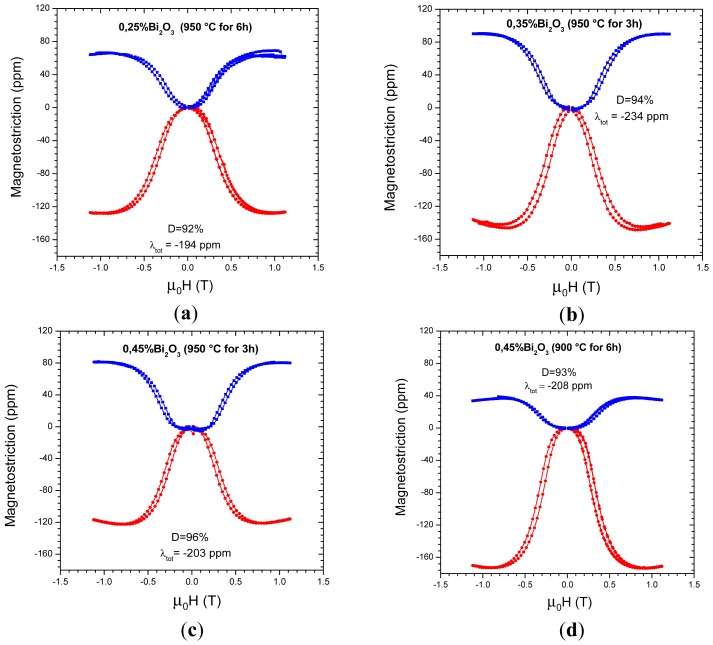
Magnetostriction of the liquid-phase sintered samples. Red curves: λ_//_; blue curves: λ_⊥_.

Figure 4 shows the magnetostriction *vs.* applied field results of the liquid-phase sintered ferrite samples. The λ_//_ values obtained are in the range from −122 ppm to −172 ppm and are in good agreement with the result of −131 to −165 ppm obtained in [13], although the present results vary within a slightly wider range. In Table 2, the values of (λ_//_)_s_, (λ_⊥_)_s_, λ_tot_ and (λ_//_)_s_ /(λ_⊥_)_s_ are compiled together. The parameter λ_tot_ of the liquid-phase sintered samples ranged from −194 to −234 ppm and the reference sample had a λ_tot_ of −228 ppm, which is within this range. The total magnetostriction λ_tot_ is directly related to the saturation magnetostriction λ_s_, which is an intrinsic material property, through the relation λ_tot_ = 3/2 λ_s_. From the results on Table 2, we conclude that the addition of 0.35% of Bi_2_O_3_ and sintering temperature of 950 °C during 3 h, resulted in the highest intrinsic magnetostriction value.

It is expected that the magnetostriction of polycrystalline samples that have isotropic microstructure with equiaxed and randomly oriented grains follow the proportion λ_//_ = −2λ_⊥_. This proportionality was not observed in all samples as a consequence of the processing method, which resulted in samples with anisotropic microstructure. The anisotropy of microstructure is more evident in the sample with 0.45% Bi_2_O_3_ sintered at 900 °C for 6 h, where λ_//_ ≈ −4.8 λ_⊥_.

The results shown in Table 2 indicate that the microstructure anisotropy is an important parameter that is affecting the linear magnetostriction. This anisotropy is also directly related to the presence of second phases, since in the sample with addition of 0.25% of Bi_2_O_3_ and sintering temperature of 950 °C during 6 h, the magnetostriction anisotropy was not present and the fraction of Bi-containing particles in the microstructure was very low. If these particles contain ferromagnetic phases, the non-uniform distribution along the sample might be the responsible for the magnetostriction anisotropy.

A more detailed study of the influence of the second phases on the magnetic properties of this ferrite would require a precise characterization of the chemical composition of such phases. It is known that the BiCo_1-x_Fe_x_O_3_ phase is paramagnetic when x = 0 (at temperatures between 100–800 K), antiferromagnetic when x = 1, and ferromagnetic at room temperature when 0.5 ≤ x ≤ 0.7 [20]. The susceptibility of this oxide is slightly field-dependent when 0.1 ≤ x ≤ 0.2 [20].

**Figure 5.**
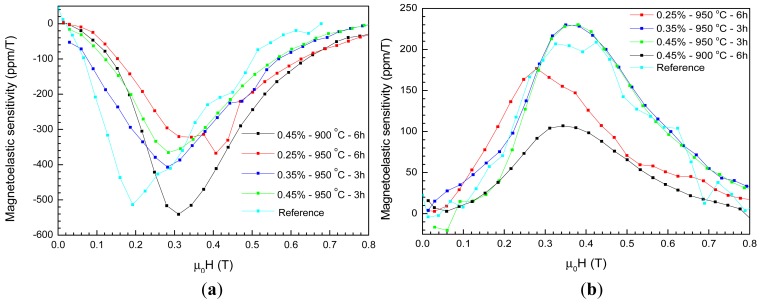
Magnetoelastic sensitivities (∂λ/∂(μ_0_H)) *vs.* applied field. (**a**) Magnetoelastic sensitivity parallel to the applied field. (**b**) Magnetoelastic sensitivity perpendicular to the applied field.

The anisotropy induced by the presence of the second phases increased considerably the linear magnetostriction and this is a very interesting characteristic for the development of force sensors, for example. Figure 5 shows the curves of the magnetoelastic sensitivities *vs.* applied field and Table 3 shows the maximum magnetoelastic sensitivity and the internal field value at which it occurs in the longitudinal and transversal modes.

As expected, the more relevant results were obtained for the direction parallel to the magnetic field, because the saturated magnetostriction values are higher in this configuration. The highest sensitivities were reached by the samples with 0.45% of Bi_2_O_3_, sintered at 900 °C for 6 h and with 0.35% of Bi_2_O_3_, sintered at 950 °C for 6 h, having the first a maximum sensitivity very close to the reference sample. Among the liquid-phase sintered samples, the one with 0.35% Bi_2_O_3_ addition presented the highest longitudinal magnetoelastic sensitivity in lower fields. Another virtue of both samples is that the maximum sensitivity occurs at low fields, lower than 0.2 T. As expected, the maximum magnetoelastic sensitivity is directly related to the maximum magnetostriction attained in the samples in the parallel configuration and therefore, must be associated with the second phases produced due to the liquid-phase sintering with Bi_2_O_3_.

**Figure 6.**
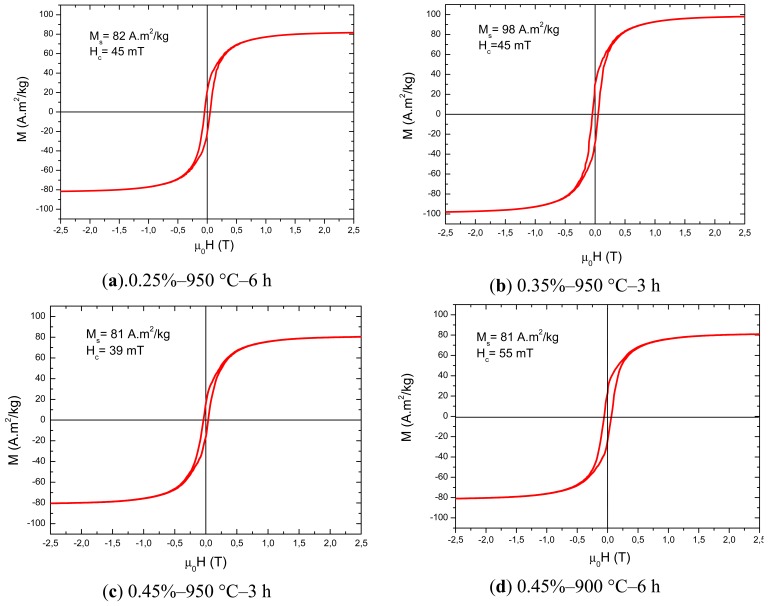
Hysteresis curves, indicating the values of M_s_ and H_c_ for each sample.

Figure 6 shows the hysteresis curves of the samples. The values of 81–82 A.m^2^/kg for the saturation magnetization (M_s_) are close to the reference sample (around 78 A.m^2^/kg) and are in good agreement with other works in literature [13,22]. The sample with 0.35% Bi_2_O_3_ presented the highest values of M_s_ and the sample with 0.45% Bi_2_O_3_, sintered at 900 °C, presented the highest coercive field (H_c_). The values of H_c_ are influenced by the fractions of second phases and porosity, as well as the grain size; the lower sintering temperature of 900 °C probably resulted in a smaller grain size, which contributed to increasing the coercivity.

## 4. Conclusions

CoFe_2_O_4_ sintered samples with relative densities higher than 91% were successfully obtained, by means of liquid-phase sintering with Bi_2_O_3_ as sintering additive. The use of sintering temperatures of 900 °C and 950 °C resulted in values of magnetostriction and magnetoelastic sensitivity as high as in a reference sample of CoFe_2_O_4_ sintered at 1,300 °C without additives. This is an important result considering that the sintering temperatures used were below 1,000 °C.

The formation of ferromagnetic second phases during liquid phase sintering, that were detected by SEM analysis, is supposed to be the responsible for the good magnetostriction and magnetoelastic sensitivity results obtained. The presence of these phases caused anisotropy of the microstructure and of the magnetostrictive behavior; however, the determination of their composition and the related magnetic properties must be confirmed.

## Figures and Tables

**Table 1.** Results of mass density measurements.

**%*Bi_2_O_3_***	***Sintering Temperature***	***Sintering Time***	***Mass Density* (*d*)** **[g/cm^3^]**	***Relative density* (*D*)** **[%]**

0.15	900 °C	3 h	3.76	71
0.15	950 °C	3 h	4.30	81
0.25	900 °C	3 h	4.16	78
0.25	950 °C	3 h	4.80	90
0.25	950 °C	6 h	4.91	92
0.35	900 °C	3 h	4.54	85
0.35	950 °C	3 h	5.02	94
0.45	900 °C	3 h	4.80	90
0.45	950 °C	3 h	5.11	96
0.45	900 °C	6 h	4.95	93

**Table 2.** Saturation values of the magnetostriction parallel and perpendicular to the magnetic field, calculated λ_tot_ (from Equation (4)), and the ratio (λ_//_)_s_/(λ_⊥_)_s_.

***Sample***	**(λ_//_)_s_** **[ppm]**	**(λ_⊥_)_s_** **[ppm]**	**λ_tot_** **[ppm]**	**(λ_//_)_s_/(λ_⊥_)_s_**

0.25%–950 °C −6 h	−128	66	−194	−1.94
0.35%–950 °C −3 h	−146	88	−234	−1.66
0.45%–950 °C −3 h	−122	81	−203	−1.51
0.45%–900 °C −6 h	−172	36	−208	−4.78
Reference	−146	76	−222	−1.92

**Table 3.** Values of maximum magnetoelastic sensitivities and the corresponding values of μ_0_H_int_.

***Sample***	***Magnetoelastic Sensitivity***			

	**| ∂λ_//_/∂(μ_0_H) | [ppm/T]**	**μ_0_H_int_ [T]**	**| ∂λ_⊥_/∂(μ_0_H) | [ppm/T]**	**μ_0_H_int_ [T]**

0.25%–950 °C–6 h	367	0.27	177	0.16
0.35%–950 °C–3 h	407	0.14	230	0.19
0.45%–950 °C–3 h	366	0.17	230	0.25
0.45%–900 °C–6 h	541	0.19	107	0.22
Reference	513	-	209	-
